# Supplementary material for: The development of blood protein profiles in extremely preterm infants follows a stereotypic evolution pattern
Source: Commun Med (Lond). 2023 Aug 2;3:107. doi: 10.1038/s43856-023-00338-1 (PMC10397184; doi:10.1038/s43856-023-00338-1)
Supplement: Supplementary file 1 — Reporting Summary [file 43856_2023_338_MOESM1_ESM.pdf]

nature portfolio

Corresponding author(s): Ann Hellström

Last updated by author(s): Jun 14, 2023

Reporting Summary

Nature Portfolio wishes to improve the reproducibility of the work that we publish. This form provides structure for consistency and transparency in reporting. For further information on Nature Portfolio policies, see our Editorial Policies and the Editorial Policy Checklist.

Statistics

For all statistical analyses, confirm that the following items are present in the figure legend, table legend, main text, or Methods section.

n/a	Confirmed	
☐	☒	The exact sample size (*n*) for each experimental group/condition, given as a discrete number and unit of measurement
☐	☒	A statement on whether measurements were taken from distinct samples or whether the same sample was measured repeatedly
☐	☒	The statistical test(s) used AND whether they are one- or two-sided<br>*Only common tests should be described solely by name; describe more complex techniques in the Methods section.*
☐	☒	A description of all covariates tested
☐	☒	A description of any assumptions or corrections, such as tests of normality and adjustment for multiple comparisons
☐	☒	A full description of the statistical parameters including central tendency (e.g. means) or other basic estimates (e.g. regression coefficient) AND variation (e.g. standard deviation) or associated estimates of uncertainty (e.g. confidence intervals)
☐	☒	For null hypothesis testing, the test statistic (e.g. *F*, *t*, *r*) with confidence intervals, effect sizes, degrees of freedom and *P* value noted<br>*Give P values as exact values whenever suitable.*
☒	☐	For Bayesian analysis, information on the choice of priors and Markov chain Monte Carlo settings
☐	☒	For hierarchical and complex designs, identification of the appropriate level for tests and full reporting of outcomes
☐	☒	Estimates of effect sizes (e.g. Cohen's *d*, Pearson's *r*), indicating how they were calculated

Our web collection on statistics for biologists contains articles on many of the points above.

Software and code

Policy information about availability of computer code

Data collection	R project for data processing
Data analysis	R project for statistical computation; packages: umap, lme4, ggplot2, tidyverse, fsmb, destiny, pheatmap, glmnet

For manuscripts utilizing custom algorithms or software that are central to the research but not yet described in published literature, software must be made available to editors and reviewers. We strongly encourage code deposition in a community repository (e.g. GitHub). See the Nature Portfolio guidelines for submitting code & software for further information.

Data

Policy information about availability of data

All manuscripts must include a data availability statement. This statement should provide the following information, where applicable:

- Accession codes, unique identifiers, or web links for publicly available datasets
- A description of any restrictions on data availability
- For clinical datasets or third party data, please ensure that the statement adheres to our policy

All summary statistics and association data are available in the Supplementary Data 1-10. Source data for figure 1, 2a and 5 are available in Source Data 1.The proteomic data of the preterm infant cohort is available in the BioStudies database (http://www.ebi.ac.uk/biostudies) under accession number S-BSST843

Human research participants

Policy information about studies involving human research participants and Sex and Gender in Research.

Reporting on sex and gender	A total of 182 extremely preterm infants born before 28 weeks of gestation from the Mega Donna Mega study were selected based on the availability of longitudinal serum samples. Altogether 105 boys and 77 girls were included. Serum samples were collected repeatedly at nine planned time points (visits) from birth to term-equivalent age, including PND1, PND3, PND7, PND14, PNW4, PMW30, PMW32, PMW36 and PMW40. The enrolled infants were classified into three groups depending on GA at birth: group 1, born at less than 25+0 (weeks+days) (N = 61); group 2, born at 25+0 to 26+6 (weeks+days) (N = 81); and group 3, born at 27+0 to 27+6 (weeks+days) of gestation (N = 40)
Population characteristics	see above
Recruitment	The current study is based on the multicenter, open-label, randomized controlled trial MegaDonnaMega (Clinical Trial.gov identifier NCT03201588). Details of the MegaDonnaMega-study are described elsewhere. In summary, infants born before 28 weeks of gestation and treated at the neonatal intensive care unit (NICU) in Gothenburg, Lund, or Stockholm, Sweden, between December 2016 and December 2019, were randomized to receive the triglyceride oil supplement Formulaid (DSM Nutritional Products Inc) containing arachidonic acid and docosahexaenoic acid or no extra supplement/standard care. Randomization was stratified according to the center and three GA groups: less than 25 weeks, 25 to 26 weeks, or 27 weeks. Twins or triplets were randomized to the same group. The supplement emulsion was administrated orally from within 72 hours of age to term-equivalent age. Written informed consent was obtained from the parents or guardians before inclusion.
Ethics oversight	The MegaDonnaMega-study followed the Consolidated Standards of Reporting Trials (CONSORT) reporting guideline. The regional ethical board of Gothenburg approved the MegaDonnaMega-study, and the Swedish Ethical Review Authority approved this extended study.

Note that full information on the approval of the study protocol must also be provided in the manuscript.

Field-specific reporting

Please select the one below that is the best fit for your research. If you are not sure, read the appropriate sections before making your selection.

☒ Life sciences ☐ Behavioural & social sciences ☐ Ecological, evolutionary & environmental sciences

For a reference copy of the document with all sections, see nature.com/documents/nr-reporting-summary-flat.pdf

Life sciences study design

All studies must disclose on these points even when the disclosure is negative.

Sample size	No priori power calculation was made. The study is the largest possible longitudinal cohort for extremely preterm infants with extensive repeated sampling and analyses.
Data exclusions	Infants from the MegaDonnaMega study with no available longitudinal blood samples up to full term age were excluded.
Replication	Blood samples were taken in line with clinical medical needs at birth, postnatal day three, seven, fourteen, twenty-eight, followed by samples taken at PMW 30, 32, 36, and term-equivalent age corresponding to 40 weeks PMA. Clinical data regarding birth, growth, nutrition and morbidities were collected prospectively according to the study protocol.
Randomization	Randomization was stratified according to the center and three GA groups: less than 25 weeks, 25 to 26 weeks, or 27 weeks. Twins or triplets were randomized to the same group.
Blinding	No blinding

Reporting for specific materials, systems and methods

We require information from authors about some types of materials, experimental systems and methods used in many studies. Here, indicate whether each material, system or method listed is relevant to your study. If you are not sure if a list item applies to your research, read the appropriate section before selecting a response.

Materials & experimental systems

n/a	Involved in the study	
☒	☐	Antibodies
☒	☐	Eukaryotic cell lines
☒	☐	Palaeontology and archaeology
☒	☐	Animals and other organisms
☐	☒	Clinical data
☒	☐	Dual use research of concern

Methods

n/a	Involved in the study	
☒	☐	ChIP-seq
☒	☐	Flow cytometry
☒	☐	MRI-based neuroimaging

Clinical data

Policy information about clinical studies

All manuscripts should comply with the ICMJE guidelines for publication of clinical research and a completed CONSORT checklist must be included with all submissions.

Clinical trial registration

NCT03201588

Study protocol

Infants born before 28 weeks of gestation and treated at the neonatal intensive care unit (NICU) in Gothenburg, Lund, or Stockholm, Sweden, between December 2016 and December 2019, were randomized to receive the triglyceride oil supplement Formulaid (DSM Nutritional Products Inc) containing arachidonic acid and docosahexaenoic acid or no extra supplement/standard care. Randomization was stratified according to the center and three GA groups: less than 25 weeks, 25 to 26 weeks, or 27 weeks. Twins or triplets were randomized to the same group. The supplement emulsion was administrated orally from within 72 hours of age to term-equivalent age.

Data collection

Infants born before 28 weeks of gestation and treated at the neonatal intensive care unit (NICU) in Gothenburg, Lund, or Stockholm, Sweden, between December 2016 and December 2019, were recruited to the study. Blood samples were taken in line with clinical medical needs at birth, postnatal day three, seven, fourteen, twenty-eight, followed by samples taken at PMW 30, 32, 36, and term-equivalent age corresponding to 40 weeks PMA. Clinical data regarding birth, growth, nutrition and morbidities were collected prospectively according to the study protocol.

Outcomes

Primary Outcome Measure:
Investigate whether enteral administration of AA and DHA in addition to commonly used regimes with parenteral olive based lipid emulsion (Clinoleic) compared to Clinoleic alone prevents the sight threatening disease Retinopathy of Prematurity (ROP). [Time Frame: When the retina is fully vascularised, i.e approximately 40 postmenstrual weeks.

Secondary Outcome measures:
1. Postnatal serum fatty acid composition in preterm infants with and without AA:DHA supplementation;
2. Postnatal brain development, as assessed by Magnetic Resonance Imaging (MRI), Volumetric and Diffusor Tensor Imaging (DTI) ;
3. Outcome in p-glucose;
4. Outcome in weight in kilograms;
5. Outcome in head circumference in centimeters;
6. Outcome in height in centimeters;
7. Outcome of neonatal morbidities. Frequency of neonatal morbidities such as bronchopulmonary dysplasia (BPD), cerebral intraventricular hemorrhage (IVH), patent ductus arteriosus (PDA), sepsis and necrotizing enterocolitis (NEC).
