# Supplementary material for: The development of blood protein profiles in extremely preterm infants follows a stereotypic evolution pattern
Source: Commun Med (Lond). 2023 Aug 2;3:107. doi: 10.1038/s43856-023-00338-1 (PMC10397184; doi:10.1038/s43856-023-00338-1)
Supplement: Supplementary file 2 — Description of Additional Supplementary Files [file 43856_2023_338_MOESM2_ESM.pdf]

Description of Additional Supplementary Files

File Name: Supplementary Data 1

Description: Clinical characteristics of the preterm infants

File Name: Supplementary Data 2

Description: Complete list of analyzed proteins

File Name: Supplementary Data 3

Description: Inter- and intra- individual variability of proteins

File Name: Supplementary Data 4

Description: Expressions of proteins in time-series clusters

File Name: Supplementary Data 5

Description: Tissue specificity of proteins in longitudinal clusters

File Name: Supplementary Data 6

Description: Immune cell specificity of proteins in longitudinal clusters

File Name: Supplementary Data 7

Description: Complete results of functional analysis of proteins in longitudinal clusters

File Name: Supplementary Data 8

Description: Variance explanation of protein levels after birth

File Name: Supplementary Data 9

Description: Blood proteins used for the predictive PNA model

File Name: Supplementary Data 10

Description: Differentially expressed proteins across GA groups

File Name: Source Data 1

Description: Source data for figure 1, figure 2a and figure 5.
