# Supplementary material for: The development of blood protein profiles in extremely preterm infants follows a stereotypic evolution pattern
Source: Commun Med (Lond). 2023 Aug 2;3:107. doi: 10.1038/s43856-023-00338-1 (PMC10397184; doi:10.1038/s43856-023-00338-1)
Supplement: Supplementary file 3 — Supplementary Information [file 43856_2023_338_MOESM3_ESM.pdf]

Supplementary Materials for

Zhong et al. The development of blood protein profiles in extremely preterm infants follows a stereotypic evolution pattern

The PDF file includes:

Supplementary Figure 1. Growth of preterm infants after birth
Supplementary Figure 2. Longitudinal clustering patterns of serum proteins after birth
Supplementary Figure 3. Longitudinal clustering patterns of serum proteins between GA groups
Supplementary Figure 4. Functional analysis of proteins in longitudinal clusters
Supplementary Figure 5. Proteins related to infant sex
Supplementary Figure 6. Proteins related to mode of delivery
Supplementary Figure 7. Protein variability explained by infant anthropometrics and perinatal factors
Supplementary Figure 8. Bland-Altman plot comparing infant predicted age and chronological ages
Supplementary Figure 9. Associations between infant postnatal standardized weight (SDS) and the delta PNA
Supplementary Figure 10. Comparisons of the effects from PNA and PMA on protein expression patterns.
Supplementary Figure 11. Overview of effects from sex and delivery mode on protein expression patterns
Supplementary Figure 12. Longitudinal blood protein profiling of preterm infants
Supplementary Figure 13. Gestational age impact on blood protein expressions after preterm birth

Other Supplementary Material for this manuscript includes the following:

Supplementary Data 1. Clinical characteristics of the preterm infants
Supplementary Data 2. Complete list of analyzed proteins
Supplementary Data 3. Inter- and intra- individual variability of proteins
Supplementary Data 4. Expressions of proteins in time-series clusters
Supplementary Data 5. Tissue specificity of proteins in longitudinal clusters

Supplementary Data 6. Immune cell specificity of proteins in longitudinal clusters

Supplementary Data 7. Complete results of functional analysis of proteins in longitudinal clusters

Supplementary Data 8. Variance explanation of protein levels after birth

Supplementary Data 9. Blood proteins used for the predictive PNA model

Supplementary Data 10. Differentially expressed proteins across GA groups

Supplementary Figure 1

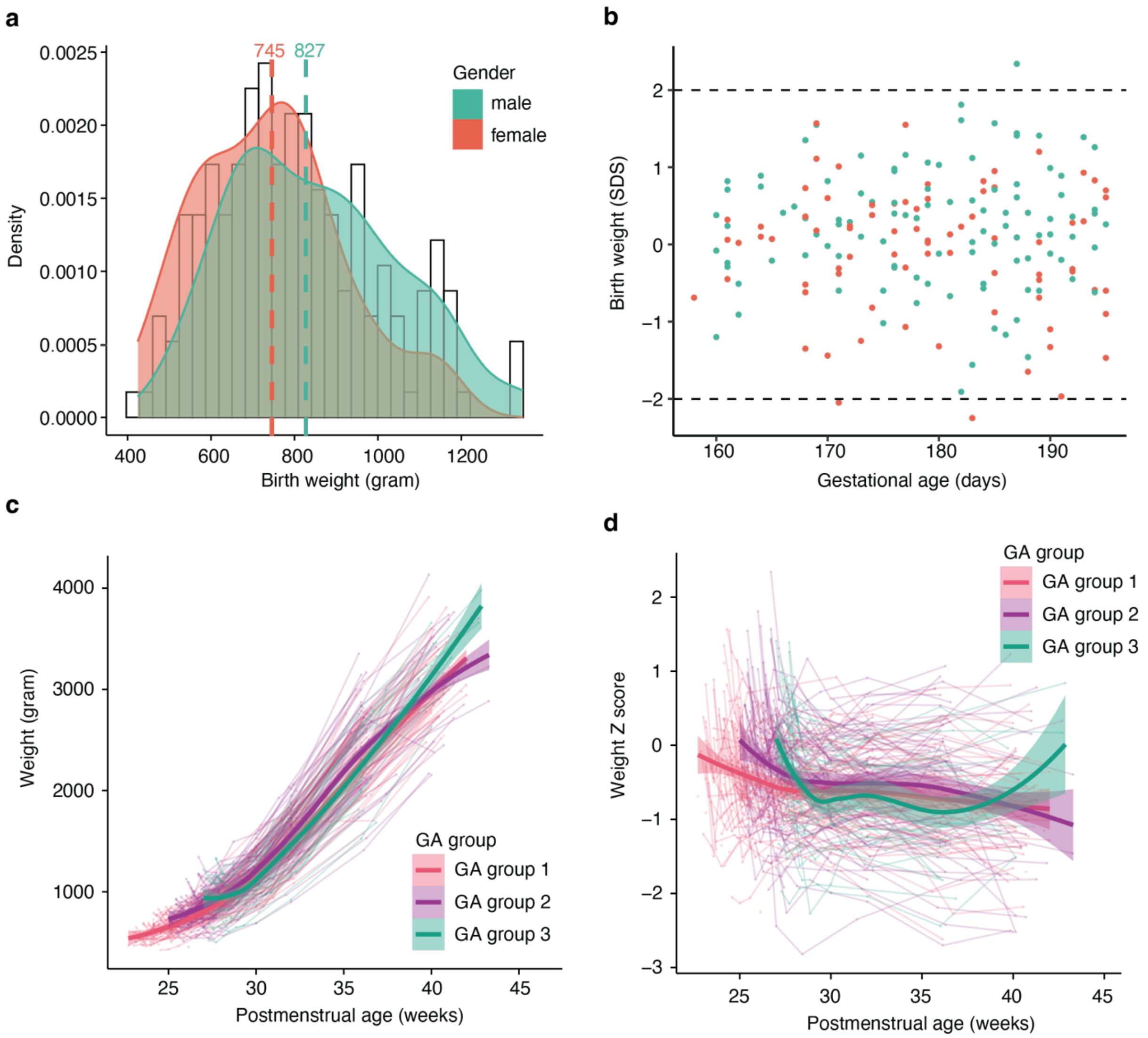

Supplementary Figure 1. Growth of preterm infants after birth. (a) A combined histogram and density plot showing the distribution of birth weight of preterm infants for male and female, respectively. The dashed lines indicate the median birth weight, color coded by males and females. (b) Scatter plot showing birth weight in standard deviations score (SDS) of all preterm infants with various gestational age. (c) Growth in weight (g) from birth to 40 weeks postmenstrual age. (d) Growth of head circumference in standard deviations score (SDS) in all preterm infants over time from birth to 40 weeks postmenstrual age.

Supplementary Figure 2

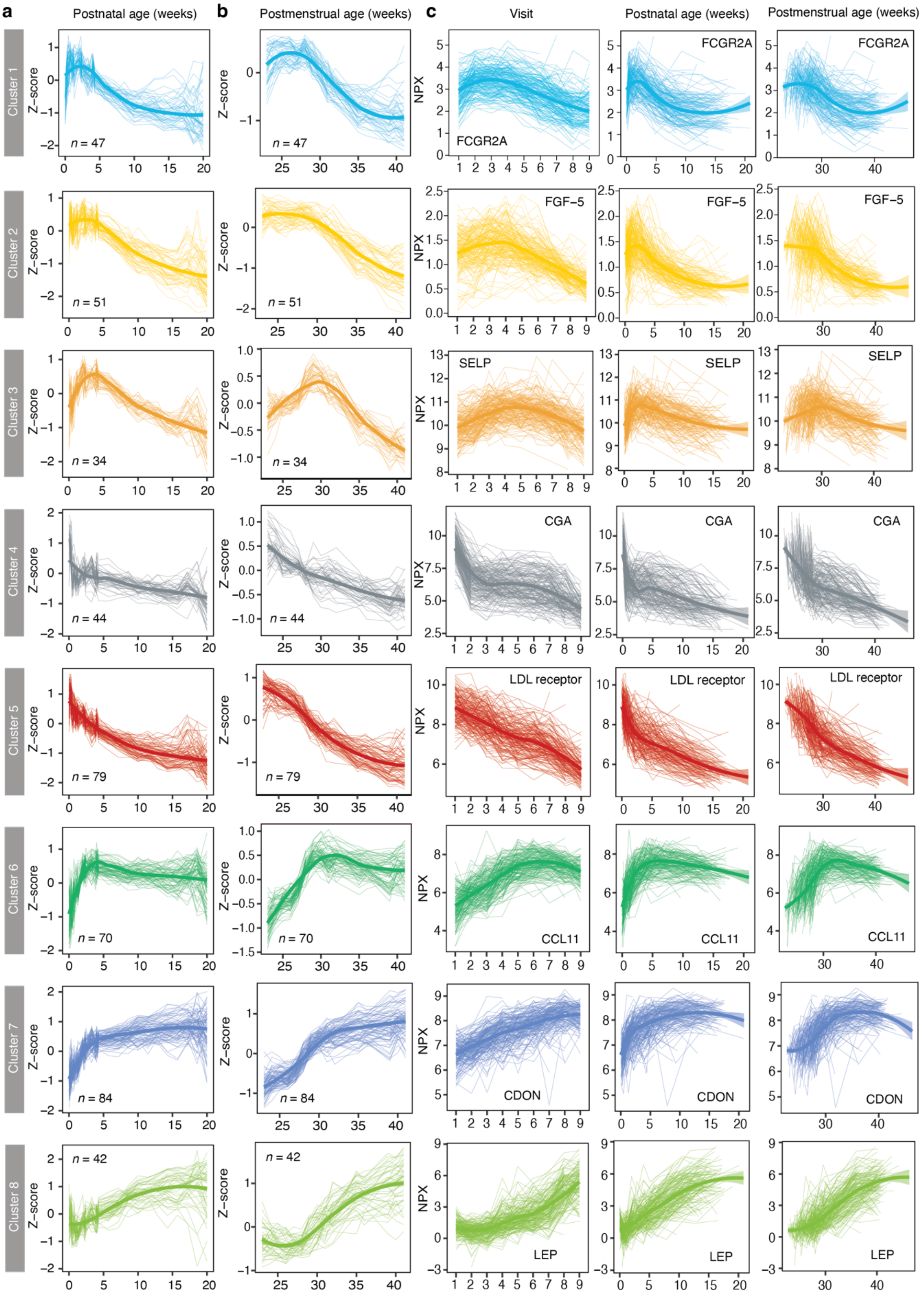

Supplementary Figure 2. Longitudinal clustering patterns of serum proteins after birth. (a) Overview of the expression profiles of all analyzed proteins in each of the eight clusters

based on infant postnatal age at sampling and mean scaled NPX values for all preterm infants (n = 182). The colored bold line represents the regression line based on all proteins and the total number of proteins in each cluster is indicated in the boxes. (b) Expression profiles of all proteins in each of the eight clusters based on infant post-menstrual age at sampling and mean scaled NPX values for all preterm infants. (c) Examples of representative protein expression patterns for one protein from each cluster with the expression level for all preterm infants (n = 182).

Supplementary Figure 3

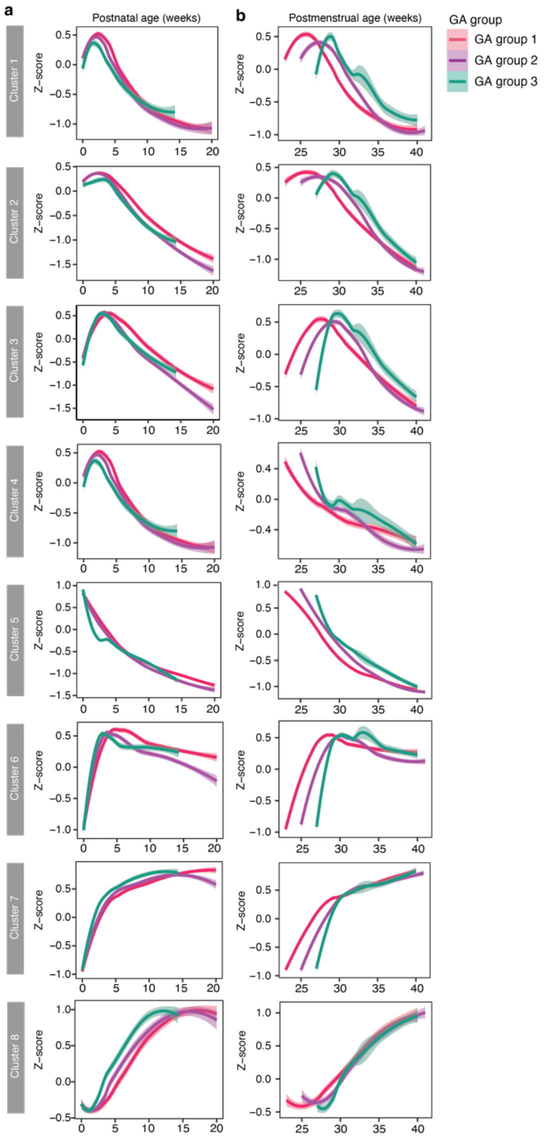

Supplementary Figure 3. Longitudinal clustering patterns of serum proteins between GA groups. (a) Expression patterns of proteins (mean scaled NPX) in each of the eight clusters based on infant postnatal age (weeks) at sampling. The color code indicates the three GA groups: group 1 (red), born at less than 25+0 (weeks+days) (N = 61); group 2 (purple),

born at 25+0 to 26+6 (weeks+days) (N = 81); and group 3 (green), born at 27+0 to 27+6 (weeks+days) of gestation (N = 40). (b) Expression patterns of proteins (mean scaled NPX) in each of the eight clusters based on infant postmenstrual age (weeks) at sampling .

Supplementary Figure 4

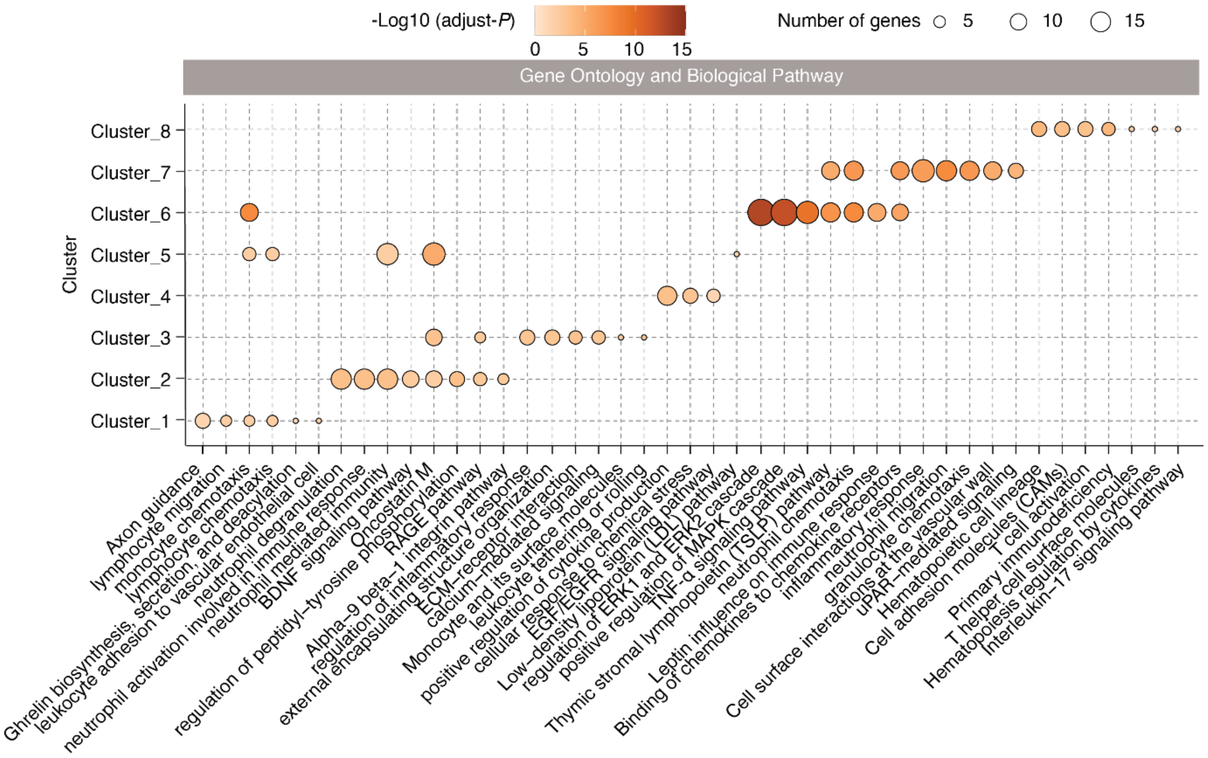

Supplementary Figure 4. Functional analysis of proteins in longitudinal clusters. Selective results of gene ontology (GO) and pathway enrichment analysis of proteins in each of eight clusters (see full details in Supplementary Data 7a and 7b). The dot size indicates the number of enriched genes in each cluster. The color code indicates the significance level (-Log_{10} adjusted *P* value) for each test (Fisher's exact test).

Supplementary Figure 5

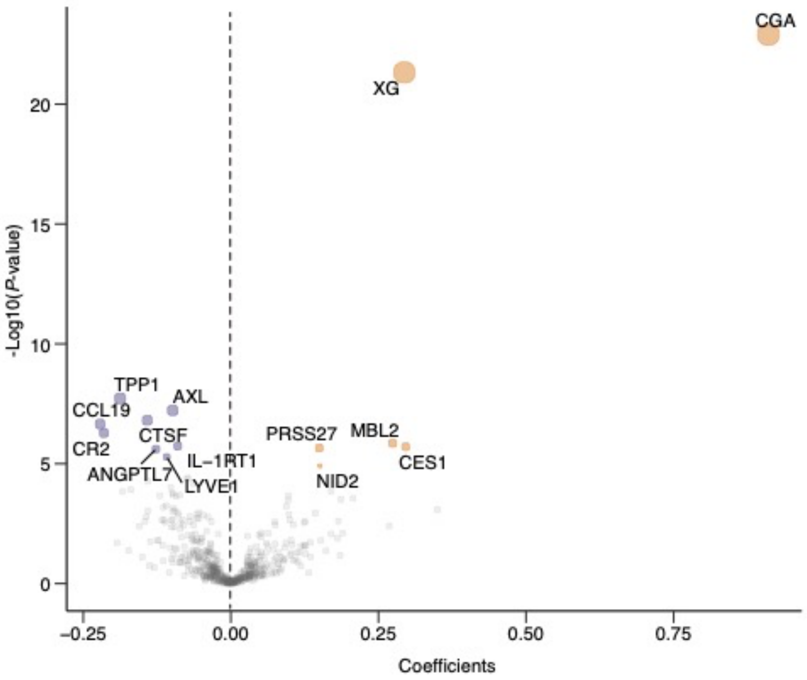

Supplementary Figure 5. Proteins related to infant sex. Volcano plot showing the sex-related proteins across the study visits. The X-axis represents the coefficients of sex. The Y-axis represents −log10 *P*-values. Differentially expressed proteins were defined as proteins with adjusted *P*-values < 0.01 (ANOVA with delivery mode and GA as covariates). Multiple test corrections have been applied to the *P*-values using the Benjamini and Hochberg method.

Supplementary Figure 6

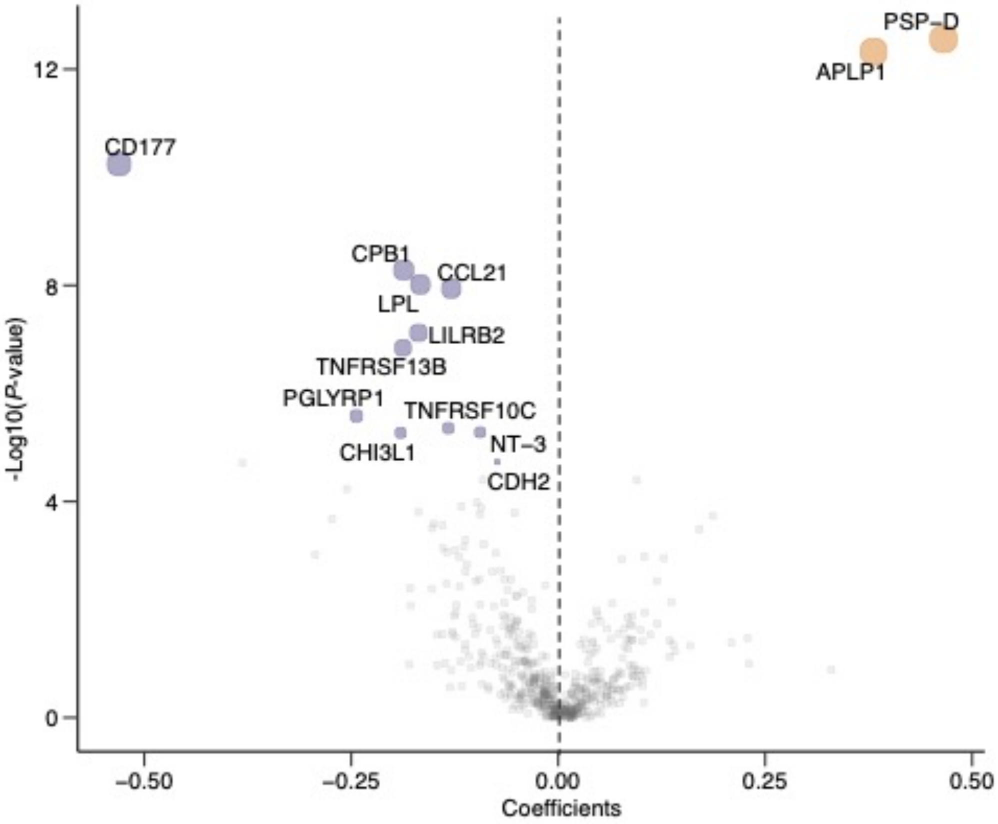

Supplementary Figure 6. Proteins related to mode of delivery. Volcano plot showing the delivery-related proteins across the study visits. The X-axis represents the coefficients of the delivery mode. The Y-axis represents −log10 *P*-values. Differentially expressed proteins were defined as proteins with adjusted *P*-values < 0.01 (ANOVA with sex and GA as covariates). Multiple test corrections have been applied to the *P*-values using the Benjamini and Hochberg method.

Supplementary Figure 7

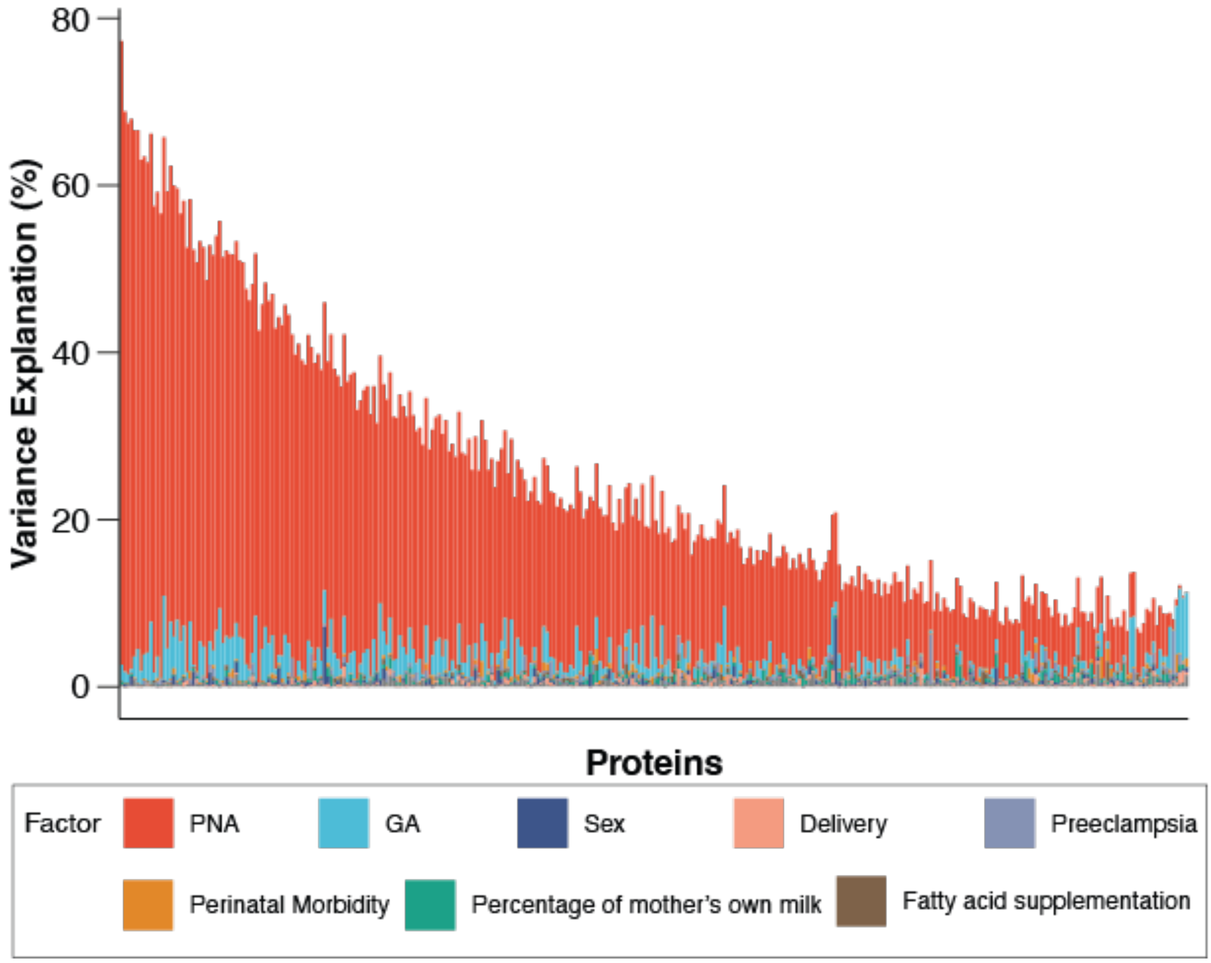

Supplementary Figure 7. Protein variability explained by infant anthropometrics and perinatal factors. Barplot showing the percentage of variance explained by postnatal age (PNA), gestational age (GA), sex, delivery mode, preeclampsia, perinatal morbidity, percentage of mother's own milk and donor milk and fatty acid supplementation for all 538 analyzed proteins.

Supplementary Figure 8

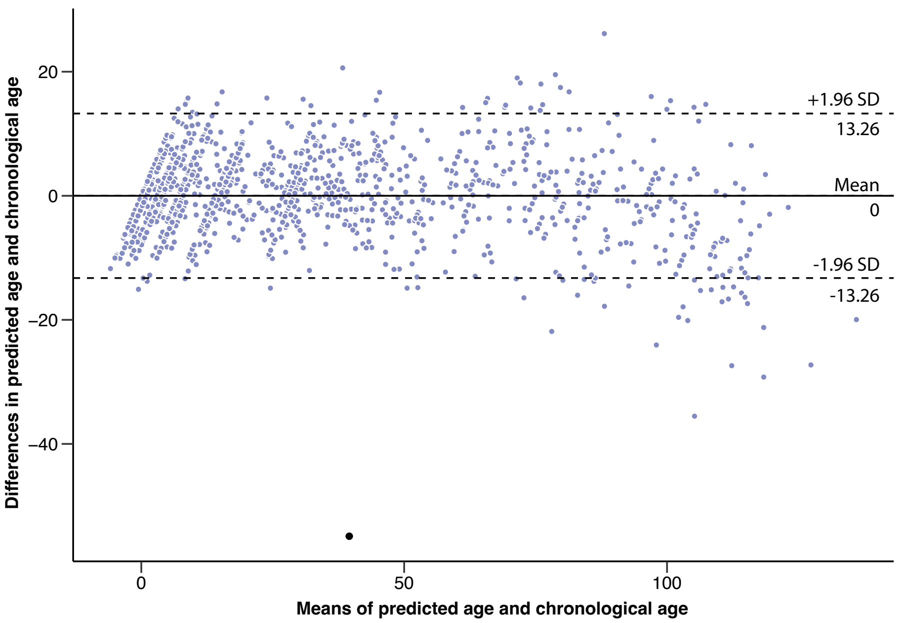

Supplementary Figure 8. Bland-Altman plot comparing infant predicted age and chronological age. The plot displays the differences between the predicted age, derived from 151 blood proteins, and the actual chronological age for 1335 blood serum samples collected from 182 preterm infants in the study. The differences are plotted against the average of the predicted and chronological ages (days).

Supplementary Figure 9

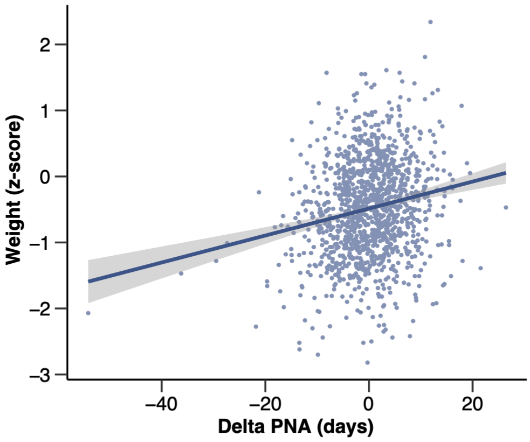

Supplementary Figure 9. Associations between infant postnatal standardized weight (SDS) and the delta PNA. The delta age (days) is determined as the difference between predicted postnatal age (PNA) and actual chronological age. A scatter plot showing the associations between standardized weight and delta age for all 1335 analyzed samples. The regression line was calculated based on a generalized linear model.

Supplementary Figure 10

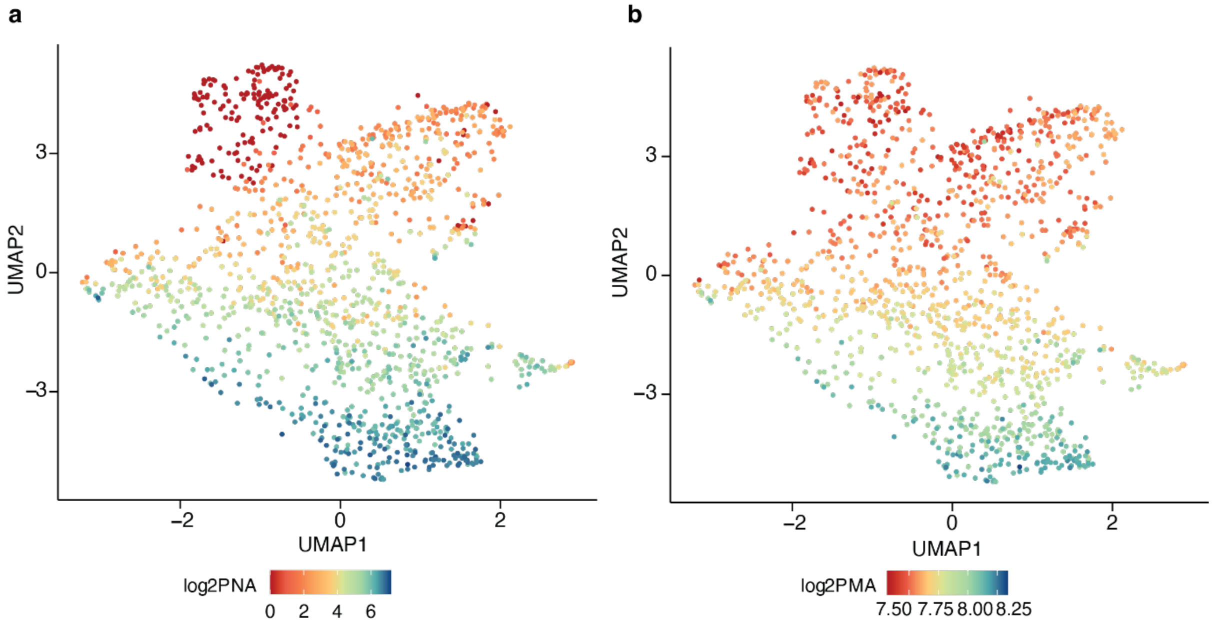

Supplementary Figure 10. Comparisons of the effects from PNA and PMA on protein expression patterns. (a) UMAP clustering results based on 1335 samples presenting the dynamic changes in blood protein expression patterns of preterm infants after birth. The color code indicates the postnatal age (Log2 days). (b) UMAP clustering results for 1335 samples from the preterm infants, color coded by postmenstrual age (Log2 days).

Supplementary Figure 11

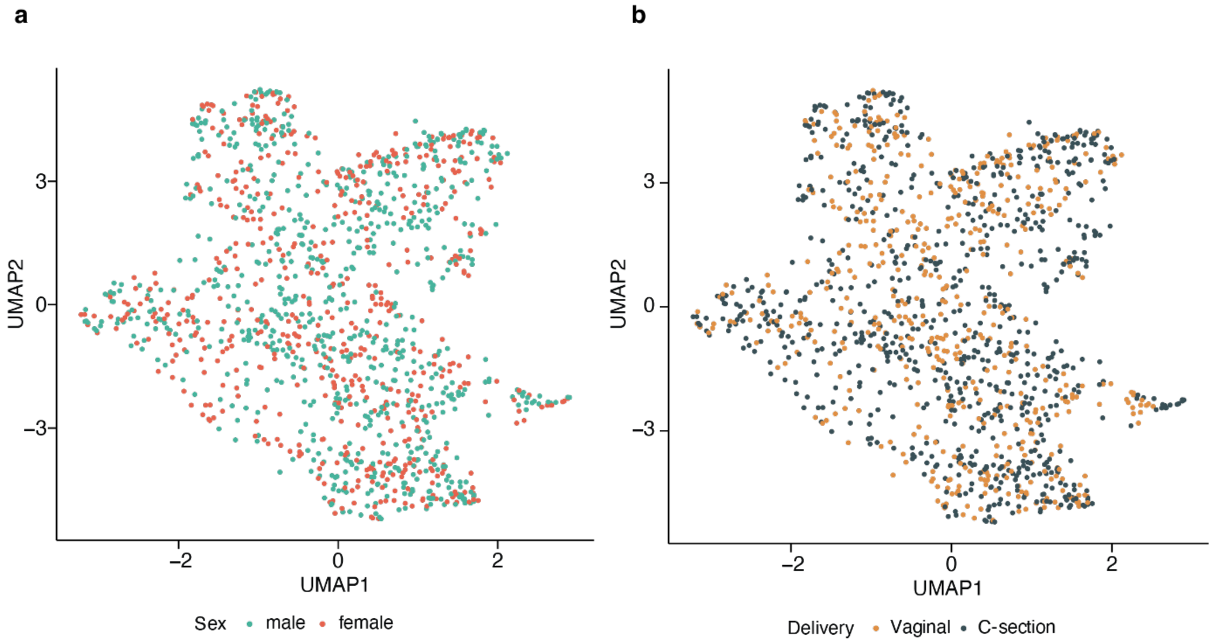

Supplementary Figure 11. Overview of effects from sex and delivery mode on protein expression patterns. (a) UMAP clustering results based on 1335 samples from preterm infants, color coded by sex. (b) UMAP clustering of preterm infants, color coded by delivery mode.

Supplementary Figure 12

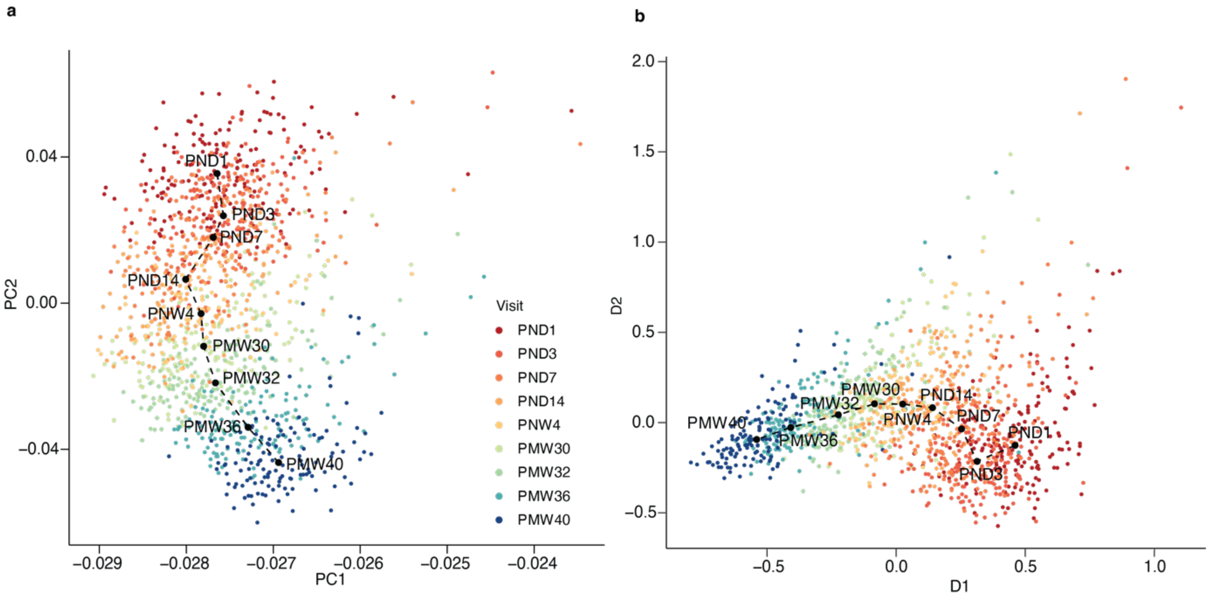

Supplementary Figure 12. Longitudinal blood protein profiling of preterm infants. (a) Principal component analysis (PCA) of the 1335 samples from preterm infants. The color code indicates the blood sampling time points. (b) Diffusion map of the 1335 samples from preterm infants.

Supplementary Figure 13

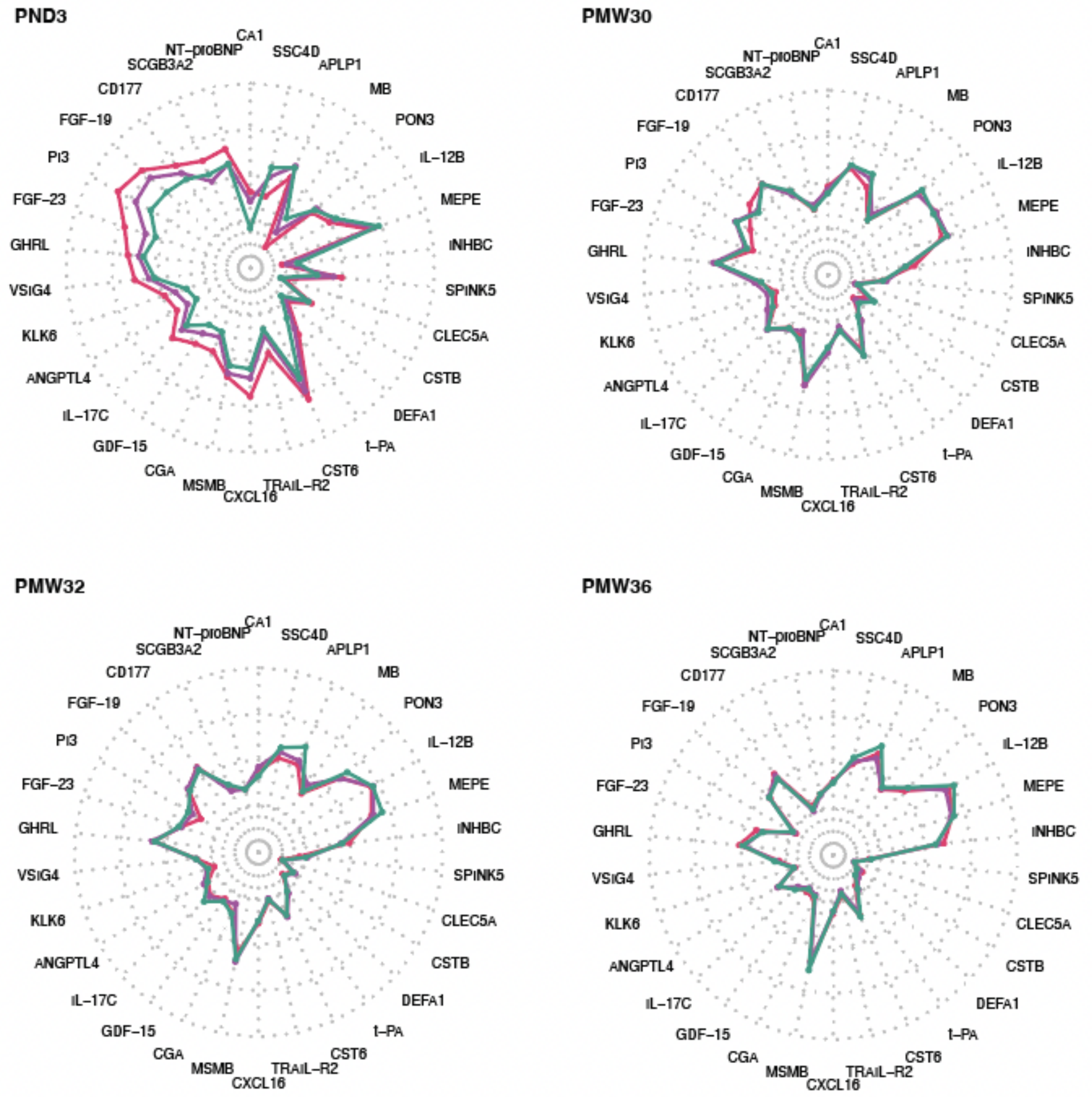

Supplementary Figure 13. Gestational age impact on blood protein expressions after preterm birth. Radar plot showing median levels of top 30 differentially expressed proteins across three GA groups at PND3, PMW30, PMW32, and PMW40. The color code indicates the three GA groups: group 1 (red), born at less than 25+0 (weeks+days) (N = 61); group 2 (purple), born at 25+0 to 26+6 (weeks+days) (N = 81); and group 3 (green), born at 27+0 to 27+6 (weeks+days) of gestation (N = 40).
